# IgG antibody response by ELISA using Wuhan and Lambda variant antigens in BBIBP-CORV vaccinated health care workers

**DOI:** 10.17843/rpmesp.2022.393.10875

**Published:** 2022-09-30

**Authors:** María García-Mendoza, Nancy Merino-Sarmiento, Gabriel De Lucio-Burga, Manuel G. Fernández-Navarro, Luis Pampa-Espinoza, Gilmer Solis-Sánchez, Maribel Huaringa-Núñez, Miryam Palomino-Rodríguez, Pamela Ríos-Monteza, Lely Solari

**Affiliations:** 1 Instituto Nacional de Salud, Lima, Peru. Instituto Nacional de Salud Lima Peru

**Keywords:** Coronavirus infections, Immunity Humoral, SARS-CoV-2 C.37 variant, Peru

## Abstract

**Objectives:**

. To evaluate the IgG antibody response by ELISA using Wuhan and Lambda antigens in health care workers with and without history of SARS-CoV-2 infection prior to immunization with the first and second doses of Sinopharm vaccine (BBIBP-CorV).

**Materials and methods:**

. An analytical study was carried out in health care workers over 18 years of age. Fifty-one participants with history and 100 participants without history of SARS-CoV-2 infection, who received two doses of Sinopharm vaccine, were included. IgG antibodies were assessed 21 days after the first dose, 21 days after the second dose and 3 months after the second dose by in-house ELISA using the complete antigen of the Wuhan variant (B.1.1) and lambda variant (C-37) of SARS-CoV-2 virus.

**Results:**

. Both groups showed a large increase in the percentage of people with antibodies after the second dose, however, this percentage decreased 3 months after the second dose. The difference between the antibody index measured by ELISA with Wuhan variant antigen versus the ELISA with lambda variant was significant (p<0.001).

**Conclusions:**

. There is a significant increase in the presence of IgG type antibodies after 15 days of the second dose of BBIBP-CorV vaccination in participants without previous infection and a decrease after 3 months of the second dose in the ratio of IgG antibody reactivity indexes in ELISAs with the variant antigen as with ELISAs with the lambda variant.

## INTRODUCTION

Since the first report at the end of 2019 in Wuhan, China, SARS-CoV-2 infection cases expanded globally to reach more than 404 million currently ^1^, of which more than 3,363,489 cases have been identified in Peruvian territory ^2^ with more than 206,000 deaths, one of the highest lethality rates in the world ^3^.

The COVID-19 pandemic has become a global public health challenge, and the main tool to counter it has been the production of vaccines, including inactivated virus vaccines ^4^. Vaccines induce an immune response that protects against the different variants of COVID-19 so far. On the other hand, it is known that previous infection also creates immunological mechanisms that protect against successive infections; however, reinfections are being reported more frequently ^5^.

Likewise, there is continuing interest in better understanding the complex relationships between the immune response elicited by infections and by SARS-CoV-2 vaccines. In addition, there is the complexity of the emerging variants of the virus and their ability to evade prior immunity ^6^. Regarding SARS-CoV-2 variants in Peru, during the second wave of infections in our country, the presence of the Gamma variant (P.1) and the wide distribution of the Lambda variant (C.37) were reported in all regions of the country ^7^ ^,^ ^8^, being the most predominant variant of interest in that wave.

The Peruvian government started the National Vaccination Program against COVID-19 in February 2021 with the inoculation of the inactivated virus vaccine BBIBP-CorV ^9^ from the Sinopharm laboratory. Priority was given to healthcare personnel due to their high exposure to SARS-CoV-2. However, there is little information on the duration of antibodies after inoculation with this vaccine. Knowing the response and duration is of great importance for implementing public health strategies regarding the application of vaccines boosters, or if it would be more useful in certain population groups with identifiable risk factors, such as certain age groups or comorbidities.

Therefore, it is important to know the response over time of IgG antibodies after the application of the inactivated virus vaccine. This study aims to evaluate the IgG antibody response by ELISA with Wuhan and Lambda variant antigens in healthcare workers vaccinated with BBIBP-COR-V.

**KEY MESSAGES**

Motivation for the study: vaccines are very important for the control of outbreaks and pandemics; however, it is necessary to evaluate their performance and duration.

Main findings: after three months of COVID-19 vaccine application, IgG antibodies decrease significantly and the reactivity index values we found were influenced by the history of infection and the presence of Lambda antigen. This study was presented on September 28, 2021 at the meeting of experts of the Peruvian Ministry of Health to evaluate the decision to apply the third dose.

Implications: it is important to continue encouraging research on immunity against COVID-19 and its application to vaccination strategies.

## MATERIALS AND METHODS

### Participants, sample size and sampling

A longitudinal analytical study was conducted. We included healthcare workers over 18 years of age, with and without a history of previous COVID-19 infection. The history of infection was determined according to the description of the participants during the blood sample collection, and was cross-checked by reviewing previous positive molecular or antigenic test results (NetLabV2 and SISCOVID).

Subjects were enrolled prior to receiving the second dose of vaccine against SARS-CoV-2 infection, 3 weeks (21 days) since the first dose, in accordance with the national vaccination policy. All participants received SARS-CoV-2 inactivated virus BBIBP-CorV vaccine from the Sinopharm laboratory. Participants who did not continue in the study were eliminated from the overall analysis.

The sample size was calculated for the mean difference with OpenEpi version 3.01. A mean of 100 ±22 in the first group and 110 ±20 in the second group ^10^, a confidence level of 95%, a power of 80% and a ratio between groups of 1 were considered as reference values. Consecutive non-probabilistic sampling was used and 151 health workers were enrolled; this enrollment did not consider *a priori* the history of previous infection, which allowed obtaining a sample with a better approximation to the real distribution in the population.

### Sample collection procedure

At the time of enrollment, study participants signed the informed consent form and provided data on sociodemographic (age and sex) and clinicopathological (presence of comorbidities) characteristics. Seven mL of venous blood were collected from each participant in tubes with clot activator, which were left at room temperature for 10 min and then centrifuged, separated by aliquots (in duplicate), coded and finally frozen at -40 °C until processing.

Sample collection was carried out from March 18 to August 6, 2021, at three points in time: 1) 21 days after the first dose of vaccine; 2) 21 days after the second dose of vaccine; and 3) 3 months after the second dose of vaccine.

### Laboratory procedure

Two Indirect ELISA (Enzyme Linked Immunosorbent Assay) tests developed in-house were used for the detection of anti-SARS-CoV-2 IgG, with Wuhan wild-type virus antigens (B) and Lambda variant (C.37), obtained from cultures in VERO-81 cells. For sample processing, plates were coated with 100 μL of antigen in carbonate-bicarbonate buffer (pH: 9.6) and placed at 4°C overnight. Subsequently, the plates were washed five times with PBS plus Tween 20 (0.05%) (PBS-T); next, 100 uL of sera and positive and negative controls diluted 1/100 in milk diluent buffer (PBS-T with 5% skim milk) were added and incubated for 1 h at 37 °C. After five washes the conjugate (mouse anti-human IgG with peroxidase) was added and incubated for 1 h at 37 °C. After the last washing step, 100 uL of 3,3’,5,5’- tetramethylbenzidine (TMB) was added to each well and were then left in a dark environment for 5 min. The colorimetric reaction was stopped with stop solution (H_2_SO_4_ 2N). The optical density (OD) was measured in an ELISA reader with 450 nm filter and 630 nm reference filter.

The cutoff value (CV) for each ELISA assay was calculated with the average of the OD values of the negative controls +3 standard deviations (SD).

We used the reactivity index (RI) (ratio of the sample OD/VC) to define the result of each sample, which was calculated for each of the samples; interpreted as reactive (RI> 1), non-reactive (RI <0.9) and indeterminate (RI: 0.9 - 1). The RI has been used in SARS-CoV-2 immunity studies as an important measurement variable to compare populations with clinical or epidemiological differences ^11^.

### Statistical analysis

Data analysis was carried out with the statistical program Stata v. 17.0 (STATA corporation, College Station, Texas, USA). During the descriptive analysis we used relative and absolute frequencies for categorical variables and medians and interquartile ranges for numerical variables. For the bivariate analysis (according to having had COVID-19 previously or not), we evaluated the difference in means using the Mann-Whitney U test. Finally, median regression was used to evaluate the association with IR.

### Ethical Aspects

The participants gave their informed consent prior to participation. This study was approved by the Institutional Research Ethics Committee of the National Institute of Health (RD-00301-2020-OGITT-INS.pdf); it has also been registered in the Health Research Projects Registry (PRISA) under code: EI00000002162.

## RESULTS

A total of 151 participants were included, of whom 100 had no history of previous SARS-CoV-2 infection; in addition, those who decided not to continue in the study were not included in the final analysis, so that complete information was available for all 151 participants. The median age was 43 years, most were adults between 30 and 59 years of age (n=113, 74.8%), 62.3% were women (n=94), and 25.8% had some comorbidity or risk condition. No statistically significant difference (p>0.05) was found in the sociodemographic or clinical characteristics between the groups with and without history of previous infection (Table 1).

**Table 1:** Characteristics of the participants, according to COVID-19 history.

Characteristics	Total N=151	Evaluated group		p-value
		No history of COVID-19 N=100	History of COVID-19 N=51	
	n (%)	n (%)	n (%)	
Age (years) ^a^	43.0 (34.0-52.0)	44.0 (33.0-54.5)	43.0 (35.0-51.0)	0.849 ^b^
Age group				
Young adult (19 - 29 years)	15 (9.9)	12 (12.0)	3 (5.9)	0.068 ^c^
Adult (30 - 59 years)	113 (74.8)	69 (69.0)	44 (86.3)	
Older adult (60 years and older)	23 (15.3)	19 (19.0)	4 (7.8)	
Sex				
Male	57 (37.7)	42 (42.0)	15 (29.4)	0.131^c^
Female	94 (62.3)	58 (58.0)	36 (70.6)	
Presence of comorbidities or at-risk condition				
No	112 (74.2)	76 (76.0)	36 (70.6)	0.472 ^c^
Yes	39 (25.8)	24 (24.0)	15 (29.4)	
Age group at risk (>60 y)	23 (15.2)	19 (19.0)	4 (7.8)	0.071 ^c^
Diabetes mellitus (Tipo 2)	6 (4.0)	5 (5.0)	1 (2.0)	0.664 ^d^
Arterial hypertension	12 (7.9)	5 (5.0)	7 (13.7)	0.107^ d^
Obesity	4 (2.6)	1 (1.0)	3 (5.9)	0.112 ^d^
Cardiopathy	1 (0.7)	0 (0.0)	1 (2.0)	0.338 ^d^
Bronchial asthma	3 (2.0)	1 (1.0)	2 (3.9)	0.264 ^d^
Cancer	3 (2.0)	2 (2.0)	1 (2.0)	1.000 ^d^
Chronic renal insufficiency	0 (0.0)	0 (0.0)	0 (0.0)	^e^
COPD	0 (0.0)	0 (0.0)	0 (0.0)	^e^

N: total number in the group, n: number in the category, IQR: interquartile range.

a Numerical variable measured as median (IQR), ^b^ Mann-Whitney U test, ^c^ Pearson’s chi-square test, ^d^ Fisher’s exact test, ^e^ p-value not calculated due to absence of cases.

The reactivity (positive result) of blood samples from subjects without previous infection was 51% after 21 days after the first dose, rising to 100% at 21 days after the second dose, and to 94% at 3 months after the second dose when exposed to the original virus antigen (B); while for the Lambda lineage (C.37) the reactivity changed to 61%, 98% and 80%, respectively. In samples from previously infected subjects, reactivity to B was 94.1%, 100% and 98%, respectively, and for C.37 it was 92.2%, 100%, and 98%, respectively (Table 2).

**Table 2:** ELISA test against antigen developed with B and C.37 lineage, according to COVID-19 history**.**

	Original Virus			Lambda (C.37)		
	21 days AFD n (%)	21 days ASD n (%)	3 months ASD n (%)	21 days AFD n (%)	21 days ASD n (%)	3 months ASD n (%)
No history of COVID-19						
Negative	31 (31.0)	0 (0.0)	3 (3.0)	17 (17.0)	0 (0.0)	2 (2.0)
Positive	51 (51.0)	100 (100.0)	94 (94.0)	61 (61.0)	98 (98.0)	80 (80.0)
Indetermined	18 (18.0)	0 (0.0)	3 (3.0)	22 (22.0)	2 (2.0)	18 (18.0)
With history of COVID-19						
Negative	2 (3.9)	0 (0.0)	0 (0.0)	0 (0.0)	0 (0.0)	1 (2.0)
Positive	48 (94.1)	51 (100)	50 (98.0)	47 (92.2)	51 (100)	50 (98.0)
Indetermined	1 (2.0)	0 (0.0)	1 (2.0)	4 (7.8)	0 (0.0)	0 (0.0)

AFD: after the first dose, ASD: after the second dose

Significant differences (p<0.001) were found between the ELISA test results of patients with no history of COVID-19 between 21 days after the first dose and 21 days after the second dose against B antigen; the results against C.37 antigen were different between all time points. No significant changes were found in the ELISA results of patients with a history of COVID-19 (p>0.05) (Table 3).

**Table 3:** Comparisons of immunogenicity determined by total IgG antibodies by ELISA against B.1.1 and C.37 antigen, according to COVID-19 background.

	Comparisons for original virus (B)			Comparisons for Lambda (C.37)			Comparison of original virus (B) and Lambda virus (C.37)		
	a vs. b^†^	b vs. c^†^	a vs. b vs. c ^††^	A vs. B^†^	B vs. C^†^	A vs. B vs. C^†^	a vs. A^‡^	b vs. B^‡^	c vs. C^‡^
No history of COVID-19	<0.001	0.050	<0.001	<0.001	<0.001	<0.001	<0.001	NC	0.010
With history of COVID-19	0.223	0.317	0.135	0.046	0.317	0.368	0.005	NC	0.020

† Stuart-Maxwell marginal homogeneity test, ††† Cochran’s Q test, ‡ Fisher’s exact test.

NC: not calculable.

Lowercase letters correspond to samples exposed to original virus (B.1.1) as follows: a=21 days after the first dose, b=21 days after the second dose, c=3 months after the second dose.

Uppercase letters correspond to samples exposed to lambda variant (C.37) as follows: A=21 days after the first dose, B=21 days after the second dose, C=3 months after the second dose.

The median RI of total IgG antibodies against lineage virus antigen was 1.6 after 21 days after the first dose; 4.8 after 21 days after the second dose and 2.7 after 3 months after the second dose; while these values for C.37 lineage antigen were 1.4, 2.6 and 1.8, respectively. The RI values were significantly higher in the group of patients with a history of previous SARS-CoV-2 infection (p<0.001) (Table 4).

**Table 4:** Changes in the reactivity index (RI) of total IgG antibodies by ELISA test against antigen and C.37, according to COVID-19 background.

Antigen	Total	No history of COVID-19 N=100	With history of COVID-19 N=51	p-value ^a^
Virus original (B.1.1)				
21 days AFD	1.6 (0.9; 7.2)	1.1 (0.9; 1.7)	8.6 (6.3; 10.1)	<0.001
21 days ASD	4.8 (3.2; 8.1)	3.7 (2.9; 5.1)	9.0 (7.0; 10.5)	<0.001
3 months ASD	2.7 (1.7; 6.1)	2.1 (1.4; 3.0)	7.2 (5.1; 9.1)	<0.001
Lambda (C.37)				
21 days AFD	1.4 (1.0; 3.8)	1.2 (1.0; 1.5)	4.9 (3.4; 5.9)	<0.001
21 days ASD	2.6 (1.9; 4.4)	2.2 (1.6; 2.8)	5.5 (3.3; 6.4)	<0.001
3 months ASD	1.8 (1.3; 4.0)	1.5 (1.1; 2.1)	4.4 (3.1; 5.7)	<0.001

Values are presented with median and interquartile range.

a Mann-Whitney U test

AFD: after the first dose, ASD: after the second dose.

The change in RI between the different time points was highly significant for both groups (with and without history of COVID-19) regardless of the antigen evaluated (B and C.37) (Figure S1 of the Supplementary Material).

Finally, we used median regression for panel data adjusted for all the variables of interest (intro selection method) and we identified that history of previous infection significantly increased the RI by 2.86; while the C.37 antigen reduced it by 1.79 with respect to B.1 .1. .1. We also found that the RI at 21 days after the second dose increased significantly by 1.31 compared to 21 days after the first dose; and it only increased by 0.43 at 3 months after the second dose compared to 21 days after the first dose (Table 5).

**Table 5:** Characteristics associated with the reactivity index (RI) of total IgG antibodies, median regression.

Characteristics	Coef_ajust_ (95% CI)	p-value
Evaluated group		
No previous infection	Reference	
With previous infection	2.86 (2.40; 3.32)	<0.001
Antigen evaluated		
Original	Reference	
Lambda (C.37)	-1.79 (-2.37; -1.21)	<0.001
Sex		
Male	Reference	
Female	0.15 (-0.05; 0.35)	0.136
Age group		
Young adult (19 - 29 years)	Reference	
Adult (30 - 59 years)	0.00 (-0.24; 0.24)	0.986
Older adult (60 years and older)	-0.19 (-0.51; 0.13)	0.246
Time of evaluation		
21 days AFD	Reference	
21 days ASD	1.31 (1.07; 1.56)	<0.001
3 months ASD	0.43 (0.22; 0.65)	<0.001

Adjusted quantile structural function model for panel data.

95% CI: 95% confidence interval.

AFD: after the first dose, ASD: after the second dose.

## DISCUSSION

It is important to carry out studies to assess the immunity status of the Peruvian population regarding the different vaccination schedules that have been administered ^12^. This study on healthcare workers immunized with the Sinopharm vaccine had this purpose. Significant changes were found in the increase of the humoral response with IgG (p<0.001) in healthcare personnel with and without previous COVID-19 infection after the application of the first and second doses of BBIBP-CorV Sinopharm vaccine against antigen B and C.37, evidencing the contribution of humoral immunity experienced by both groups. Xia *et al*. reported that BBIBP-CorV Sinopharm vaccine applied within 21 days caused a considerable increase in the neutralizing antibody titer ^13^.

The Sinopharm vaccine has a reported efficacy of 78.1% against COVID-19 infection ^14^, but this efficacy could have decrease over time according to the different variants of SARS-CoV-2 ^15^. In our study, we evidenced a significant decrease (p<0.001) in the humoral response three months after the second dose of BBIBP-CorV vaccine was administered to healthcare personnel with and without previous COVID-19 infection against antigen B and C.37. Jeewandara *et al*. reported a decrease in the antibody response three months after the second dose of BBIBP-CorV vaccine was administered in all age groups ^16^. Levin *et al*. reported that the level of neutralizing antibodies decreases rapidly during the first three months, with a relatively slow decline ^17^. Dorian-Rose *et al*. also reported a decrease in RI levels during follow-up; however, this generally occurs after six months ^18^; this data coincides with our results. In Peru, a study conducted after our sampling took place also found a decrease in humoral immunity in 252 health care workers, where only 47.32% presented neutralizing antibodies 180 days after follow-up ^19^.

The variants also determine the level of immune response to be evaluated ^20^. Our study evaluated the response to ELISA developed with antigen of the Lambda variant, which was the predominant one during the second wave. We observed reactivity, although it showed a lower RI than those produced with the original virus (B) in immunized persons ^21^. The COVID-19 variants are presenting more and more mutations and changes in their structure with respect to the wild Wuhan virus ^22^. The Delta and Omicron variants present more mutations only in the spike protein, which largely explains the evasion of previous immunity obtained by vaccines or previous infection ^23^. 

Our study highlights the need to monitor vaccinated persons over months. In Peru, the third dose of the vaccine has been applied generally using combinations of different platforms, which is necessary in order to evaluate the new circulating variants. This will allow us to have a better picture of when it will be necessary to administer new vaccine doses in the future.

The main limitation of this study was the restricted inclusion of participants with no history of previous infection, which did not allow adequate comparison of two populations with similar proportions. Another limitation was the poor adherence of some participants who decided not to continue due to a personal decision, which is why we decided not to include them in the overall analysis. Venipuncture and serial intakes were described as the cause for not continuing or withdrawing from the study. Another limitation was the non-measurement of cellular immunity, which is an important part of the immune defense system and could explain the lack of correlation between the effectiveness of the vaccines in preventing mild or severe infection and death.

In conclusion, we found a significant increase in the humoral response after the first and second doses of Sinopharm’s vaccine as measured by indirect ELISA developed in-house, but after the third month, this response decreased significantly. These results are important to implement in vaccination strategies for the COVID-19 booster dose.
