# Supplementary material for: IgG antibody response by ELISA using Wuhan and Lambda variant antigens in BBIBP-CORV vaccinated health care workers
Source: Rev Peru Med Exp Salud Publica. 2022 Sep 30;39(3):267–73. doi: 10.17843/rpmesp.2022.393.10875 (PMC11397685; doi:10.17843/rpmesp.2022.393.10875)
Supplement: Supplementary material. — Available in the electronic version of the RPMESP. [file rpmesp-39-03-10875-s001.docx]

**MATERIAL SUPLEMENTARIO**


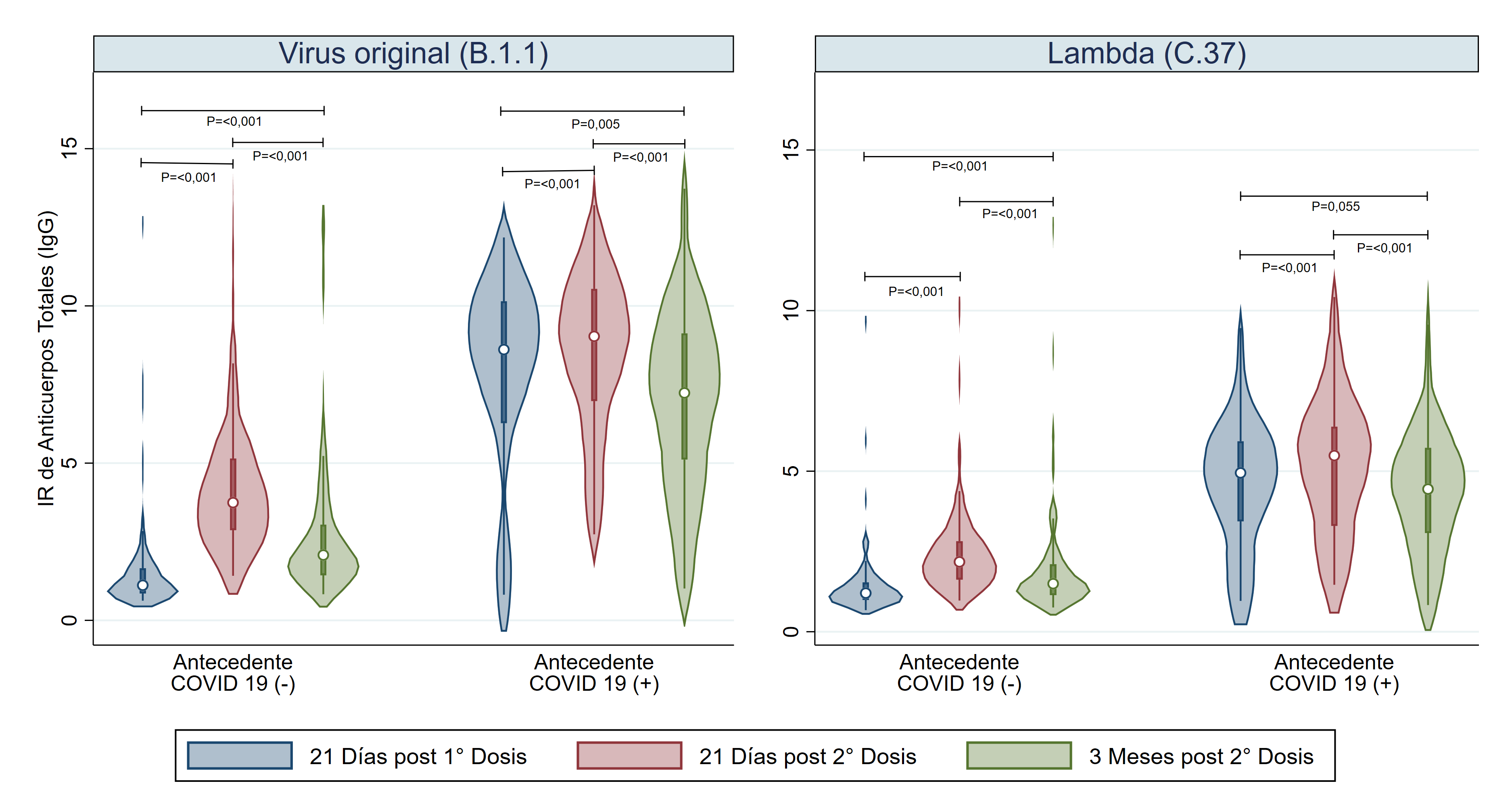


**Figura S1.** Gráfico de violín de los cambios en el índice de reactividad (IR) de anticuerpos totales IgG ante antígeno B.1.1 y C.37, según antecedentes de COVID-19 (comparaciones mediante prueba signos rangos de Wilcoxon).
