# Supplementary material for: IgG antibody response by ELISA using Wuhan and Lambda variant antigens in BBIBP-CORV vaccinated health care workers
Source: Rev Peru Med Exp Salud Publica. 2022 Sep 30;39(3):267–73. doi: 10.17843/rpmesp.2022.393.10875 (PMC11397685; doi:10.17843/rpmesp.2022.393.10875)
Supplement: Translation

# Translation: Anticuerpos IgG determinados mediante ELISA desarrollados con antígenos de linajes Wuhan y Lambda en trabajadores de salud vacunados con BBIBP-CORV

**Authors:** María García-Mendoza, Nancy Merino-Sarmiento, Gabriel De Lucio-Burga, Manuel G. Fernández-Navarro, Luis Pampa-Espinoza, Gilmer Solis-Sánchez, Maribel Huaringa-Núñez, Miryam Palomino-Rodríguez, Pamela Ríos-Monteza, Lely Solari

**Affiliations:** 1 Instituto Nacional de Salud, Lima, Peru.

**Keywords:** Infecciones por coronavirus, Inmunidad Humoral, Variante SARS-CoV-2 C.37, Perú

## Abstract

**Objetivos.:**

Evaluar la respuesta de anticuerpos IgG determinada mediante ELISA utilizando antígenos de los linajes Wuhan y Lambda en trabajadores de la salud con y sin antecedente de infección por SARS-CoV-2 previa a la inmunización con la primera y segunda dosis de la vacuna Sinopharm (BBIBP-CorV).

**Materiales y métodos.:**

Se realizó un estudio analítico en trabajadores de salud mayores de 18 años. Se incluyeron 51 participantes con antecedente y 100 participantes sin antecedente de infección por SARS-CoV-2, quienes recibieron dos dosis de la vacuna Sinopharm. Los anticuerpos IgG se evaluaron 21 días después de la primera dosis, 21 días después de la segunda dosis y 3 meses después de la segunda dosis mediante una prueba de ELISA *in house* desarrollado utilizando el antígeno completo del linaje Wuhan (B) y del linaje Lambda(C.37) del virus de SARS-CoV-2.

**Resultados.:**

En ambos grupos se observó un incremento del porcentaje de personas con anticuerpos luego de la segunda dosis, sin embargo, este porcentaje disminuyó luego de 3 meses de la segunda dosis. Se halló una diferencia significativa entre el índice de anticuerpos medido por ELISA con el antígeno del linaje Wuhan versus el ELISA con el linaje Lambda (p<0,001).

**Conclusiones.:**

Existe un aumento significativo de la presencia de anticuerpos tipo IgG luego de 15 días de la segunda dosis de la vacunación con BBIP-CORV en los participantes sin infección previa y una disminución, luego de 3 meses de la segunda dosis, de la razón de índices de reactividad de anticuerpos IgG en los ELISA desarrollados con el antígeno de la variante como en los ELISA desarrollados con la variante Lambda.

## INTRODUCCIÓN

Desde su primer reporte a fines del 2019 en Wuhan, China, los casos de infección por SARS-CoV-2 se expandieron a nivel global hasta alcanzar más de 404 millones en la actualidad ^1^, de ellos, en territorio peruano se han identificado más de 3 363 489 casos ^2^ con más de 206 mil muertes, una de las tasas más altas de letalidad en el mundo ^3^.

La pandemia por COVID-19 se convirtió en un desafío para la salud pública a nivel mundial, y la principal herramienta para combatirla han sido la generación de vacunas, incluyendo las de virus inactivado ^4^. Las vacunas inducen respuesta inmune que protege contra las distintas variantes de la COVID-19 hasta ahora. Por otro lado, se conoce que la infección previa también genera mecanismos inmunológicos que nos salvaguardan contra infecciones sucesivas; sin embargo, las reinfecciones se están reportando con más frecuencia ^5^. 

Asimismo, continúa el interés por comprender mejor las complejas relaciones entre la respuesta inmune provocada por infecciones y por las vacunas contra el SARS-CoV-2. A todo esto se le agrega la complejidad de las variantes emergentes del virus y la capacidad que tienen para evadir la inmunidad previa ^6^. En lo referente a las variantes del SARS-CoV-2 en el Perú, durante la segunda ola de contagios en nuestro país, se reportó la presencia de la variante Gamma (P.1) y la amplia distribución de la variante Lambda (C.37) identificada en todas las regiones del país ^7^ ^,^ ^8^, siendo la variante de interés más predominante en dicha ola. 

El gobierno peruano inició el Programa Nacional de Vacunación contra la COVID-19 en febrero del 2021 con la inoculación de la vacuna BBIBP-CorV ^9^ de virus inactivado del laboratorio Sinopharm; se priorizó al personal sanitario debido a su alta exposición al SARS-CoV-2. Sin embargo, existe poca información sobre la duración de los anticuerpos luego de la inoculación con esta vacuna. Conocer la respuesta y la duración tiene una gran importancia para implementar estrategias en salud publica en la aplicación de los *booster* en la población, o si tendría más utilidad en ciertos grupos poblacionales con factores de riesgo identificables, como pueden ser ciertos grupos de edad o por comorbilidades. 

Por lo tanto, es importante conocer la respuesta en el tiempo de los anticuerpos de tipo IgG luego de la aplicación de la vacuna de virus inactivado. El presente estudio tiene como objetivo evaluar la respuesta de anticuerpos IgG mediante ELISA con antígenos de variante Wuhan y Lambda en trabajadores de salud vacunados con BBIBP-COR-V.

**MENSAJE CLAVE**

Motivación para realizar el estudio: las vacunas son muy importantes para el control de brotes y pandemias, sin embargo, es necesario evaluar su rendimiento y duración.

Principales hallazgos: posterior a los tres meses de la aplicación de la vacuna contra la COVID-19, los anticuerpos IgG disminuyen significativamente y los valores de índices de reactividad hallados se han visto influenciados por los antecedentes de infección y la presencia del antígeno Lambda. Este estudio fue presentado el 28 de septiembre del 2021 en la junta de expertos del Ministerio de Salud del Perú para evaluar la decisión de aplicar la tercera dosis.

Implicancias: es importante continuar incentivando las investigaciones sobre la inmunidad en COVID-19 y su aplicación en las estrategias de vacunación.

## MATERIALES Y MÉTODOS

### Participantes, tamaño muestral y muestreo

Se realizó un estudio analítico longitudinal en el cual se incluyó a trabajadores de salud mayores de 18 años, con y sin antecedentes de infección previa de COVID-19; el antecedente de infección se determinó según descripción de los participantes durante la obtención de la muestra de sangre, y se cotejó mediante la revisión de los resultados previos de prueba molecular o antigénica positiva (NetLabV2 y SISCOVID). 

Los sujetos fueron enrolados previo a recibir la segunda dosis de la vacuna contra la infección por SARS-CoV-2, habiendo transcurrido 3 semanas (21 días) desde la primera dosis, de acuerdo con la política de vacunación nacional. Todos los participantes recibieron vacuna BBIBP-CorV de virus inactivado SARS-CoV-2 del laboratorio Sinopharm. Los participantes que no prosiguieron en el estudio fueron eliminados del análisis global. 

El tamaño muestral fue calculado para la diferencia de medias con OpenEpi versión 3.01. Se consideró como valor referencial una media de 100 ±22 en el primer grupo y de 110 ±20 en el segundo ^10^, un nivel de confianza del 95%, una potencia de 80% y una razón entre grupos de 1. Se determino un tamaño muestral mínimo de 140 personas. Se empleó un muestreo no probabilístico consecutivo enrolándose a 151 trabajadores de salud, dicho enrolamiento no contempló *a priori* los antecedentes de infección previa, lo cual permitió obtener una muestra con mayor aproximación a la distribución real en la población.

### Procedimiento de recolección de muestra

Al momento del enrolamiento, los participantes del estudio firmaron el consentimiento informado, además se obtuvieron datos de las características sociodemográficas (edad y sexo) y clínico patológicas (presencia de comorbilidades). De cada participante se recolectó 7 mL de sangre venosa en tubos con activador de coágulo, y se dejó a temperatura ambiente por 10 min para luego centrifugar, separar por alícuotas (por duplicado), codificar y finalmente congelar a -40 °C hasta su procesamiento.

Esta obtención se realizó desde el 18 de marzo al 06 de agosto del 2021, en tres momentos: 1) luego de 21 días de la primera dosis de la vacuna; 2) luego de 21 días de la segunda dosis de la vacuna y 3) luego de 3 meses de la segunda dosis de la vacuna.

### Procedimiento de laboratorio

Se utilizaron dos pruebas de ELISA (Enzyme Linked Immunosorbent Assay) Indirecta desarrollada *in house* para la detección de IgG anti-SARS-CoV-2, con antígenos del virus salvaje de Wuhan (B) y variante Lambda (C.37), obtenidos de cultivos en células VERO - 81. Para el procesamiento de las muestras se recubrieron las placas con 100 μL de antígeno en *buffer* carbonato-bicarbonato (pH: 9,6) y se colocaron a 4 °C durante toda la noche. Posteriormente, las placas fueron lavadas cinco veces con PBS más *Tween* 20 (0,05%) (PBS-T); seguidamente, se adicionó 100 uL de sueros y controles positivos y negativos diluidos 1/100 en *buffer* diluyente de leche (PBS-T con 5% de leche descremada) y se incubó durante 1 h a 37 °C. Después de cinco lavados se agregó el conjugado (IgG antihumano de ratón con peroxidasa) y se incubó durante 1 h a 37 °C. Luego del último paso de lavado se añadió a cada pozo 100 uL de 3,3’,5,5’- tetrametilbencidina (TMB) y se dejó reposar en un ambiente oscuro durante 5 min. La reacción colorimétrica se detuvo con solución de parada (H_2_SO_4_ 2N). La densidad óptica (DO) fue medida en un lector de ELISA con filtro de 450 nm y con filtro de referencia de 630 nm.

El valor de corte (VC) para cada ensayo de ELISA fue calculado con el promedio de los valores de DO de los controles negativos +3 desviaciones estándar (DE).

Para definir el resultado de cada muestra se utilizó el índice de reactividad (IR) (relación de la muestra DO/VC) el cual se calculó para cada una de las muestras; interpretándose como reactivo (IR> 1), no reactivo (IR <0.9) e indeterminado (IR: 0,9 - 1). El IR ha sido utilizado en estudios de inmunidad del SARS-CoV-2 como una variable importante de medición para comparar poblaciones con diferencias clínicas u epidemiológicas ^11^.

### Análisis estadístico

El análisis de datos se llevó a cabo con el programa estadístico Stata v. 17.0 (STATA corporation, College Station, Texas, USA). Para el análisis descriptivo se utilizaron frecuencias relativas y absolutas para variables categóricas y medianas y rangos intercuartílicos para variables numéricas. Para el análisis bivariado, (de acuerdo con haber tenido COVID-19 previamente o no) se evaluó la diferencia de medias mediante la prueba U de Mann-Whitney. Por último, se realizó una regresión de medianas para evaluar la asociación con el IR. 

### Aspectos éticos

Los participantes dieron su consentimiento informado previo a su participación. El estudio cuenta con la aprobación del Comité Institucional de Ética en Investigación del Instituto Nacional de Salud (RD-00301-2020-OGITT-INS.pdf); asimismo, ha sido ingresado en el Registro de Proyectos de Investigación de Salud (PRISA) mediante el código: EI00000002162.

## RESULTADOS

Se incluyeron 151 participantes, de los cuales 100 no tenían antecedente de infección previa por SARS-CoV-2; además, no se incluyeron en el análisis final a los que decidieron no continuar en el estudio, por lo que se contó con información completa de los 151 participantes. La mediana de la edad fue de 43 años, a predominio de adultos de entre 30 a 59 años (n=113, 74,8%), el 62,3% fueron mujeres (n=94), y el 25,8% presentó alguna comorbilidad o condición de riesgo. No se encontró diferencia estadísticamente significativa (p>0,05) en las características sociodemográficas o clínicas entre los grupos con y sin antecedentes de infección previa (Tabla 1).

**Tabla 1:** Características de las personas incluidas en el estudio, según antecedentes de COVID-19.

Características	Total N=151	Grupo evaluado		Valor de p
		Sin antecedentes de COVID-19 N=100	Con antecedentes de COVID-19 N=51	
	n (%)	n (%)	n (%)	
Edad (en años) ^a^	43,0 (34,0-52,0)	44,0 (33,0-54,5)	43,0 (35,0-51,0)	0,849 ^b^
Grupo etario				
Adulto joven (19 - 29 años)	15 (9,9)	12 (12,0)	3 (5,9)	0,068 ^c^
Adulto (30 - 59 años)	113 (74,8)	69 (69,0)	44 (86,3)	
Adulto mayor (60 años a más)	23 (15,3)	19 (19,0)	4 (7,8)	
Sexo				
Masculino	57 (37,7)	42 (42,0)	15 (29,4)	0,131^c^
Femenino	94 (62,3)	58 (58,0)	36 (70,6)	
Presencia de comorbilidades o condición de riesgo				
No	112 (74,2)	76 (76,0)	36 (70,6)	0,472 ^c^
Sí	39 (25,8)	24 (24,0)	15 (29,4)	
Grupo etario de riesgo (>60 a)	23 (15,2)	19 (19,0)	4 (7,8)	0,071 ^c^
Diabetes mellitus (Tipo 2)	6 (4,0)	5 (5,0)	1 (2,0)	0,664 ^d^
Hipertensión arterial	12 (7,9)	5 (5,0)	7 (13,7)	0,107^ d^
Obesidad	4 (2,6)	1 (1,0)	3 (5,9)	0,112 ^d^
Cardiopatía	1 (0,7)	0 (0,0)	1 (2,0)	0,338 ^d^
Asma bronquial	3 (2,0)	1 (1,0)	2 (3,9)	0,264 ^d^
Cáncer	3 (2,0)	2 (2,0)	1 (2,0)	1,000 ^d^
Insuficiencia renal crónica	0 (0,0)	0 (0,0)	0 (0,0)	^e^
EPOC	0 (0,0)	0 (0,0)	0 (0,0)	^e^

N: cantidad total en el grupo, n: cantidad en la categoría, RIC: rango intercuartil.

a Variable numérica que se mide en mediana (RIC), ^b^ prueba U de Mann-Whitney, ^c^ prueba chi cuadrado de Pearson, ^d^ prueba exacta de Fisher, ^e^ valor de p no calculable por ausencia de casos.

La reactividad (resultado positivo) de las muestras de sangre de los sujetos sin infección previa fue de 51% luego de 21 días de la primera dosis, subiendo a 100% a los 21 días luego de la segunda dosis, y a 94% a los 3 meses luego de la segunda dosis cuando se expuso al antígeno del virus original (B); mientras que para el linaje Lambda (C.37) la reactividad cambio de 61%, 98% y 80%, respectivamente. En las muestras de sujetos con infección previa la reactividad a B fue de 94,1%, 100% y 98%, respectivamente mientras que ante C.37 fue de 92,2%, 100%, y 98%, respectivamente (Tabla 2).

**Tabla 2:** Prueba de ELISA ante antígeno desarrollado con linaje B y C.37, según antecedentes de COVID-19.

	Virus original			Lambda (C.37)		
	21 días LPD n (%)	21 días LSD n (%)	3 meses LSD n (%)	21 días LPD n (%)	21 días LSD n (%)	3 meses LSD n (%)
Sin antecedente de COVID-19						
Negativo	31 (31,0)	0 (0,0)	3 (3,0)	17 (17,0)	0 (0,0)	2 (2,0)
Positivo	51 (51,0)	100 (100,0)	94 (94,0)	61 (61,0)	98 (98,0)	80 (80,0)
Indeterminado	18 (18,0)	0 (0,0)	3 (3,0)	22 (22,0)	2 (2,0)	18 (18,0)
Con antecedente de COVID-19						
Negativo	2 (3,9)	0 (0,0)	0 (0,0)	0 (0,0)	0 (0,0)	1 (2,0)
Positivo	48 (94,1)	51 (100)	50 (98,0)	47 (92,2)	51 (100)	50 (98,0)
Indeterminado	1 (2,0)	0 (0,0)	1 (2,0)	4 (7,8)	0 (0,0)	0 (0,0)

LPD: luego de la primera dosis, LSD: luego de la segunda dosis

Se encontraron diferencias significativas (p<0,001) entre los resultados de la prueba de ELISA de pacientes sin antecedentes de COVID-19 entre los 21 días luego de la primera dosis y 21 días luego de la segunda dosis ante antígeno B; los resultados ante antígeno C.37 fueron diferentes entre todos los momentos. No se encontraron cambios significativos en los resultados de la prueba de ELISA de pacientes con antecedentes de COVID-19 (p>0,05) (Tabla 3).

**Tabla 3:** Comparaciones de la inmunogenicidad determinada por anticuerpos totales IgG mediante prueba de ELISA ante antígeno B.1.1 y C.37, según antecedentes de COVID-19.

	Comparaciones para virus original (B)			Comparaciones para Lambda (C.37)			Comparación de virus original (B) y Lambda (C.37)		
	a vs. b^†^	b vs. c^†^	a vs. b vs. c ^††^	A vs. B^†^	B vs. C^†^	A vs. B vs. C^†^	a vs. A^‡^	b vs. B^‡^	c vs. C^‡^
Sin antecedente de COVID-19	<0,001	0,050	<0,001	<0,001	<0,001	<0,001	<0,001	NC	0,010
Con antecedente de COVID-19	0,223	0,317	0,135	0,046	0,317	0,368	0,005	NC	0,020

† Prueba de homogeneidad marginal de Stuart-Maxwell, ^††^ prueba Q de Cochran, ^‡^ prueba exacta de Fisher.

NC: no calculable.

Las letras en minúscula corresponden a muestras expuesta a virus original (B.1.1) de la siguiente manera: a=21 días luego de la primera dosis, b=21 días luego de la segunda dosis, c=3 meses luego de la segunda dosis.

Las letras en mayúsculas corresponden a muestras expuesta a variante lambda (C.37) de la siguiente manera: A=21 días luego de la primera dosis, B=21 días luego de la segunda dosis, C=3 meses luego de la segunda dosis.

Las medianas del IR de los anticuerpos totales IgG ante antígeno con virus de linaje fue de 1,6 luego de 21 días de la primera dosis; 4,8 luego de 21 dias de la segunda y 2,7 luego de 3 meses de la segunda dosis; mientras que estos valores ante antígeno con linaje C.37 fueron de 1,4; 2,6 y 1,8, respectivamente. Los valores de IR fueron significativamente mayores en el grupo de pacientes con antecedentes de infección previa por SARS-CoV-2 (p<0,001) (Tabla 4). El cambio en el IR entre los diferentes momentos fue altamente significativo para ambos grupos (con y sin antecedentes de COVID-19) sin distinción del antígeno evaluado (B y C.37) (Figura S1 del Material Suplementario).

**Tabla 4:** Cambios del índice de reactividad (IR) de anticuerpos totales IgG mediante prueba de ELISA ante antígeno y C.37, según antecedentes de COVID-19.

Antígeno	Total	Sin antecedente de COVID-19 N=100	Con antecedente COVID-19 N=51	Valor de p ^a^
Virus original (B.1.1)				
21 días LPD	1,6 (0,9; 7,2)	1,1 (0,9; 1,7)	8,6 (6,3; 10,1)	<0,001
21 días LSD	4,8 (3,2; 8,1)	3,7 (2,9; 5,1)	9,0 (7,0; 10,5)	<0,001
3 meses LSD	2,7 (1,7; 6,1)	2,1 (1,4; 3,0)	7,2 (5,1; 9,1)	<0,001
Lambda (C.37)				
21 días LPD	1,4 (1,0; 3,8)	1,2 (1,0; 1,5)	4,9 (3,4; 5,9)	<0,001
21 días LSD	2,6 (1,9; 4,4)	2,2 (1,6; 2,8)	5,5 (3,3; 6,4)	<0,001
3 meses LSD	1,8 (1,3; 4,0)	1,5 (1,1; 2,1)	4,4 (3,1; 5,7)	<0,001

Los valores se presentan con la mediana y rango intercuartílico

a Prueba U de Mann-Whitney

LPD: luego de la primera dosis, LSD: luego de la segunda dosis

Por último, se hizo una regresión de medianas para datos en panel ajustado por todas las variables de interés (método de selección intro) donde se identificó que los antecedentes de infección previa incrementan significativamente el IR en 2,86; mientras que el antígeno C.37 lo reduce en 1,79 respecto al B.1.1. Se evidenció, además, que el IR a los 21 días luego de la segunda dosis respecto a los 21 días luego de la primera aumentaba significativamente en 1,31; mientras que a los 3 meses luego de la segunda dosis respecto a los 21 días luego de la primera solo aumentaba en 0,43 (Tabla 5).

**Tabla 5:** Características asociadas al índice de reactividad (IR) de anticuerpos totales IgG, regresión de medianas.

Características	Coef_ajust_ (IC 95%)	Valor de p
Grupo evaluado		
Sin infección previa	Referencia	
Con infección previa	2,86 (2,40; 3,32)	<0,001
Antígeno evaluado		
Original	Referencia	
Lambda (C.37)	-1,79 (-2,37; -1,21)	<0,001
Sexo		
Masculino	Referencia	
Femenino	0,15 (-0,05; 0,35)	0,136
Grupo etario		
Adulto joven (19 - 29 años)	Referencia	
Adulto (30 - 59 años)	0,00 (-0,24; 0,24)	0,986
Adulto mayor (60 años a más)	-0,19 (-0,51; 0,13)	0,246
Momento de evaluación		
21 días LPD	Referencia	
21 días LSD	1,31 (1,07; 1,56)	<0,001
3 meses LSD	0,43 (0,22; 0,65)	<0,001

Modelo ajustado de funciones estructurales de cuantiles para datos en panel.

IC 95%: intervalo de confianza al 95%.

LPD: luego de la primera dosis, LSD: luego de la segunda dosis

## DISCUSIÓN

Es importante realizar estudios que permitan valorar el estado de la inmunidad en la población peruana frente a los distintos esquemas de vacunas que se han venido administrando ^12^. El presente estudio realizado en trabajadores de salud inmunizados con la vacuna de Sinopharm tuvo este propósito. Se encontraron cambios significativos en el aumento de la respuesta humoral con IgG (p<0,001) en personal de salud con y sin infección previa de COVID-19 tras la aplicación de la primera y segunda dosis de vacuna BBIBP-CorV Sinopharm ante el antígeno B y C.37, evidenciando el aporte de la inmunidad humoral que experimentan ambos grupos. Xia *et al.* reportaron que la vacuna de BBIBP-CorV Sinopharm aplicada entre 21 días provocaba un aumento considerable del título de anticuerpos neutralizantes ^13^. 

La vacuna de Sinopharm ha reportado una eficacia de 78,1% frente a infección por COVID-19^14^, pero esta eficacia podría haber ido disminuyendo en el tiempo de acuerdo con las distintas variantes del SARS-CoV-2 ^15^. En nuestro estudio se evidencio una disminución significativa (p<0,001) de la respuesta humoral luego de tres meses de colocada la segunda dosis de la vacuna de BBIBP-CorV en personal de salud con y sin infección previa de la COVID-19 ante el antígeno B y C.37. Jeewandara *et al.* reportaron una disminución de la respuesta de anticuerpos luego de tres meses de la colocación de la segunda dosis de vacuna de BBIBP-CorV en todos los grupos de edad ^16^. Levin *et al*. reportaron que el nivel de anticuerpos neutralizantes disminuye rápidamente durante los primeros tres meses, con un descenso relativamente lento ^17^. Dorian-Rose *et al.* también reportaron una disminución en los niveles de los IR durante el seguimiento; sin embargo, esto generalmente ocurre luego de seis meses ^18^, datos que coinciden con nuestro estudio. En Perú, un estudio realizado en una fecha posterior a nuestro muestreo observó también una disminución de la inmunidad humoral en 252 trabajadores de salud, donde solo el 47,32% presentaron anticuerpos neutralizantes 180 días después del seguimiento ^19^. 

Las variantes también determinan el nivel de respuesta inmune que se evalúa ^20^. Nuestro estudio evaluó la respuesta a ELISA desarrollado con antígeno de la variante Lambda, que fue la predominante durante la segunda ola. Se encontró presencia de reactividad, aunque en personas inmunizadas tuvo un IR más bajo de aquellos producidos con el virus original (B) ^21^. Las variantes de la COVID-19 están reportando cada vez más mutaciones y cambios en su estructura con respecto al virus salvaje de Wuhan ^22^, pues las variantes Delta y Ómicron presentan más mutaciones solo en la proteína espiga, ello explica en gran parte la evasión de la inmunidad previa obtenida por vacunas o infección previa ^23^. 

El presente estudio resalta la necesidad de monitorizar a las personas vacunadas a lo largo de los meses. En el Perú se ha aplicado la terceras dosis de la vacuna generalmente utilizando combinaciones de distintas plataformas, lo cual es necesario evaluar de manera particular con las nuevas variantes circulantes. Esto permitirá tener un mejor panorama sobre el momento en el que será necesario colocar nuevas dosis de vacunas a nuestra población en el futuro. 

La principal limitante de este estudio fue la restringida inclusión de participantes sin antecedentes de infección previa, lo cual no permitió comparar adecuadamente dos poblaciones con proporciones similares. Otra limitante fue la poca adherencia de algunos participantes que por decisión personal decidieron no continuar, por ello se decidió no incluirlos en el análisis global. La venopunción y tomas seriadas se describieron como la causa para no continuar o retiro del estudio. Otra de las limitantes fue la no medición de la inmunidad celular que forma parte importante del sistema de defensa inmune en las personas y podría explicar la falta de correlación entre efectividad de las vacunas para prevenir infección leve o infección severa y muerte. 

En conclusión, se observa un aumento importante de la respuesta humoral luego de la colocación de la primera y segunda dosis de la vacuna de Sinopharm medido a través de ELISAS desarrolladas indirecto *in house*, pero de manera posterior al tercer mes, esta respuesta disminuye significativamente. Estos resultados son importantes para implementarlos en las estrategias de vacunación para la dosis de refuerzo contra la COVID-19.
